# Elevated plasma miRNA-122, -140-3p, -720, -2861, and -3149 during early period of acute coronary syndrome are derived from peripheral blood mononuclear cells

**DOI:** 10.1371/journal.pone.0184256

**Published:** 2017-09-29

**Authors:** Xiang-Dong Li, Yue-Jin Yang, Lai-Yuan Wang, Shu-Bin Qiao, Xiang-Feng Lu, Yong-Jian Wu, Bo Xu, Hong-Fan Li, Dong-Feng Gu

**Affiliations:** 1 Department of Cardiology, Fuwai Hospital, National Center for Cardiovascular Diseases, Chinese Academy of Medical Sciences and Peking Union Medical College, Beijing, China; 2 Department of Epidemiology, State Key Laboratory of Cardiovascular Disease, Fuwai Hospital, National Center for Cardiovascular Diseases, Chinese Academy of Medical Sciences and Peking Union Medical College, Beijing, China; University of Louisville, UNITED STATES

## Abstract

**Objective:**

Our previous study has found that circulating microRNA (miRNA, or miR) -122, -140-3p, -720, -2861, and -3149 are significantly elevated during early stage of acute coronary syndrome (ACS). This study was conducted to determine the origin of these elevated plasma miRNAs in ACS.

**Methods:**

qRT-PCR was performed to detect the expression profiles of these 5 miRNAs in liver, spleen, lung, kidney, brain, skeletal muscles, and heart. To determine their origins, these miRNAs were detected in myocardium of acute myocardial infarction (AMI), and as well in platelets and peripheral blood mononuclear cells (PBMCs, including monocytes, circulating endothelial cells (CECs) and lymphocytes) of the AMI pigs and ACS patients.

**Results:**

MiR-122 was specifically expressed in liver, and miR-140-3p, -720, -2861, and -3149 were highly expressed in heart. Compared with the sham pigs, miR-122 was highly expressed in the border zone of the ischemic myocardium in the AMI pigs without ventricular fibrillation (P < 0.01), miR-122 and -720 were decreased in platelets of the AMI pigs, and miR-122, -140-3p, -720, -2861, and -3149 were increased in PBMCs of the AMI pigs (all P < 0.05). Compared with the non-ACS patients, platelets miR-720 was decreased and PBMCs miR-122, -140-3p, -720, -2861, and -3149 were increased in the ACS patients (all P < 0.01). Furthermore, PBMCs miR-122, -720, and -3149 were increased in the AMI patients compared with the unstable angina (UA) patients (all P < 0.05). Further origin identification revealed that the expression levels of miR-122 in CECs and lymphocytes, miR-140-3p and -2861 in monocytes and CECs, miR-720 in monocytes, and miR-3149 in CECs were greatly up-regulated in the ACS patients compared with the non-ACS patients, and were higher as well in the AMI patients than that in the UA patients except for the miR-122 in CECs (all P < 0.05).

**Conclusion:**

The elevated plasma miR-122, -140-3p, -720, -2861, and -3149 in the ACS patients were mainly originated from CECs and monocytes.

## Introduction

Our previous study has found that high expression levels of plasma miR-122, -140-3p, -720, -2861, and -3149 are helpful for improving the discrimination of acute coronary syndrome (ACS) from non-ACS patients, and miR-122 and -3149 may be potential biomarkers for early diagnosis of ACS [1]. However, the origin of these miRNAs are still unclear.

It is well known that platelets and peripheral blood mononuclear cells (PBMCs, including circulating endothelial cells, monocytes, and lymphocytes) play important roles in the development of atherosclerosis to ACS. Risk factors, such as hyperlipidemia, hyperglycemia, and smoke, result in injury and dysfunction of endothelial cells, abnormality of lipid metabolism, and activation of monocytes by oxidized low density lipoprotein (ox-LDL). The activated monocytes migrate under the subendothelial space and differentiate into macrophages, which then take up ox-LDL and convert into foam cells [2]. These foam cells can in turn secrete proinflammatory factors, inducing the apoptosis of mononuclear macrophages, stimulating vascular proliferation, and consequently promoting the development of atherosclerosis [3]. The narrowed atherosclerotic coronary with abnormal vasodilatation results in myocardial ischemia and anoxia, that is angina pectoris. The injured endothelial cells and mononuclear macrophages release tissue factors, elastolytic enzyme, and matrix metalloproteinase, thus contributing to the thinning and rupture of fibrous cap. The released cholesterol crystal, ox-LDL, mononuclear macrophages, and foam cells cooperatively stimulate endothelial cells, platelets, and acute thrombotic events, leading to unstable angina (UA) or acute myocardial infarction (AMI) [4].

Thus, atherosclerosis, coronary heart disease (CHD), and ACS are a series of events, based on the damage of vascular endothelial cells and activation of monocytes and platelets. In this pathophysiological process, injury of vascular endothelial cells is the initial step, activation of monocytes is the promotional step, and activation of platelets is the trigger step, whereas myocardial infarction is just the result of coronary occulusion. Therefore, we hypohesized that the elevated plasma miRNAs during ACS, especially miR-122 and -3149 that increased early after coronary occulusion and before myocardial necrosis, might be originated from PBMCs or platelets but not myocardial cells. The aim of the present study was to determine the origin of the elevated plasma miR-122, -140-3p, -720, -2861, and -3149 during ACS.

## Methods

### Animal experimental protocols

Animals and animal experimental protocols were described previously [1]. In summary, thirty six Bama male minipigs (10 months old) weighing 25 to 35 kg were fed a high-cholesterol diet without limitation of activities. Then pigs were randomly assigned to Sham (n = 6) and AMI groups (n = 30), and the AMI animals were further divided into AMI with ventricular fibrillation (VF, AMI-VF) and AMI without VF (AMI-NVF) groups after the operation. At the end of the procedure, peripheral blood samples (15 mL) was collected into EDTA-containing tubes. After the animals were sacrificed by an injection of 15% KCl (1 ml/kg), tissue samples were collected from liver, spleen, lung, kidney, brain, skeletal muscles, and myocardium of the non-ischemic, border, and ischemic areas. Tissue samples were rinsed by phosphate-buffered saline (PBS) to clear the residual blood, and then were directly put into liquid nitrogen for further analysis.

All animals received humane care in compliance with the Guide for the Care and Use of Laboratory Animals published by the National Institutes of Health, USA. The animal experimental protocols and procedures were approved by the Care of Experimental Animals Committee of FUWAI Hospital, National Center for Cardiovascular Diseases, Chinese Academy of Medical Sciences and Peking Union Medical College, China (Approval NO. 2012-1-48-973).

### Population

To insure that all of the participants were finally diagnosed according to coronary angiography, 36 patients without CHD (non-CHD), 44 stable angina (SA), 65 UA, and 53 AMI patients were selected according to the previous described criteria [5, 6] in the cardiac catheterization laboratory in FUWAI Hospital, National Center for Cardiovascular Diseases, Beijing, China, between February 2012 and August 2012. After puncture of the peripheral artery, the first 5 mL of peripheral blood were discarded, and then 15 mL of peripheral blood samples were collected into EDTA-containing tubes from the patients.

The protocol of this study was approved by the Ethics Committee of FUWAI Hospital, National Center for Cardiovascular Diseases, Chinese Academy of Medical Sciences and Peking Union Medical College, China (Approval NO. 2011–341). Written informed consent was obtained from each patient before enrolment.

### Isolation of circulating platelets and peripheral blood mononuclear cells

Peripheral blood samples (15 mL) from animals or patients were diluted and mixed with equal volume of PBE buffer solution (containing 10% fetal bovine serum, EDTA 2mM, and PBS, pH 7.3–7.4, 4°C). The diluted blood samples were layered onto an equal volume of Percoll suspension (specific gravity = 1.077 g/mL), and were centrifuged at 2,000 × g for 30 min at 4°C. The centrifuged blood samples were divided into 4 layers, the supernatant was rich in platelets, the second layer contained PBMCs, the third layer was the Percoll suspension, and the bottom layer was the red and white blood cells. The supernatant and the second layer were carefully transferred into a new tube, and were further centrifuged at 2000 × g for 15 minutes at 4°C. The supernatant was collected and was further centrifuged at 4000 × g for 15 minutes at 4°C to pellet the platelets, while the remnants was further centrifuged at 2000 × g for 15 minutes at 4°C to pellet PBMCs.

After three times of washing with PBE solution, the pelleted human PBMCs were resuspended with 80μL PBE solution, and were incubated with 20μL CD-146 magnetic beads (Miltenyi Biotec, Germany) for 15 minutes at 4°C. After three times of washing with 2 mL PBE buffer, the PBMCs were resuspended with 0.5 mL PBE buffer. Then, the cell suspension was applied onto the column placed in the magnetic field, and the CD-146 labeled circulating endothelial cells (CECs) were collected. The CECs-depleted suspension, that containing monocytes and lymphocytes, was re-centrifuged at 2000 × g for 15 minutes at 4°C to pellet the remaining mononuclear cells. The remaining mononuclear cells were resuspended with 80μL PBE solution, and were incubated with 20μL CD-14 magnetic beads (Miltenyi Biotec, Germany) for 15 minutes at 4°C. The CD-14 labeled monocytes were collected after passing through the column placed in the magnetic field, and the left suspension was re-centrifuged at 2000 × g for 15 minutes at 4°C to pellet the remaining lymphocytes. The isolated platelets, PBMCs, monocytes, CECs, and lymphocytes were stored at −80°C.

### RNA preparation

Total RNA was isolated from platelets, monocytes, CECs, lymphocytes, and tissue samples with TRIzol Reagent (Invitrogen, USA) according to the manufacturer’s protocol. In brief, total RNA was purified and ultimately eluted into 20 μL of RNase-free water. RNA quantity was assessed by NanoDrop (NanoDrop Products, Wilmington, Del., USA).

### Quantitative real-time polymerase chain reaction analysis

The expression levels of specific miRNAs were analyzed by miRNA stem loop quantitative real-time polymerase chain reaction (qRT-PCR) technology on an ABI PRISM7900 system according to the protocol of the manufacturer (Applied Biosystems, USA). TaqMan qRT-PCR was carried out with FastStart Universal Probe Master Mix (Roche, Germany) to detect the expression levels of miR-122, -140-3p, -720, and -1228 (Assay ID: 002245, 002234, 002895, and 002919, respectively; Applied Biosystems, USA). qRT-PCR was carried out with Fast SYBR Green Select Master Mix (Applied Biosystems, USA) to detect the expression levels of miR-2861 and -3149 (miR-2861: RT primer CTCAACTGGTGTCGTGGAGTCGGCAATTCAGTTGAGCCGCCCAC, forward primer ACACTCCAGCTGGGGGGGCCTGGCGGT, and reverse primer TGGTGTCGTGGAGTCG; miR-3149: RT primer CTCAACTGGTGTCGTGGAGTCGGCAATTCAGTTGAGATACACAC, forward primer ACACTCCAGCTGGGTTTGTATGGATATGTGT, and reverse primer TGGTGTCGTGGAGTCG; Nanjing BioSteed BioTechnologies Co., Ltd, China).

cDNA was synthesized from 20 ng of total RNA using a cDNA synthesis kit (Roche, Germany) at 16°C for 30 min, 42°C for 30 min, and denaturation of the enzyme at 85°C for 5 min. The assay was run in 20 μL triplicate reactions for each case to allow for assessment of technical variability. Briefly, the reactions were incubated at 50°C for 2 min, 95°C for 10 min, followed by 45 cycles of 95°C for 15 s, 60°C for 60 s. The relative expression levels for each miRNA were computed using the comparative CT method (ΔΔCt). Of note is that miR-122 was not detected in many PBMCs samples even after 45 cycles of real-time PCR. Therefore, the Ct values from qRT-PCR assays greater than 45 were treated as 45. Ct values were converted into copy numbers (copy no. = 2 (−Ct)) using a standard curve, and normalized to miR-1228 (ΔCt = Ct miR-X − Ct miR-1228) [7]. The data obtained by qRT-PCR were translated into log2 (relative level), and the outliers were excluded using scatterplot during statistical analysis.

### Statistical analysis

Patients’ baseline characteristics of different groups were compared by oneway ANOVA. For the data obtained by qRT-PCR, a widely used method to present relative expression of miRNA is the 2^-ΔΔct^ method. All values of miRNAs are expressed as mean ± SD. Comparisons of parameters among ≥ 3 groups were analyzed by oneway ANOVA, followed by post hoc testing with Bonferroni correction. Independent-sample T test was used for 2 group comparisons. All P values were two-sided and differences were considered statistically significant at a value of P < 0.05. All statistical calculations were performed by the SPSS 20.0.

## Results

### MiRNAs expression profiles in heart, liver, spleen, lung, kidney, brain, and skeletal muscles

**Fig 1:**
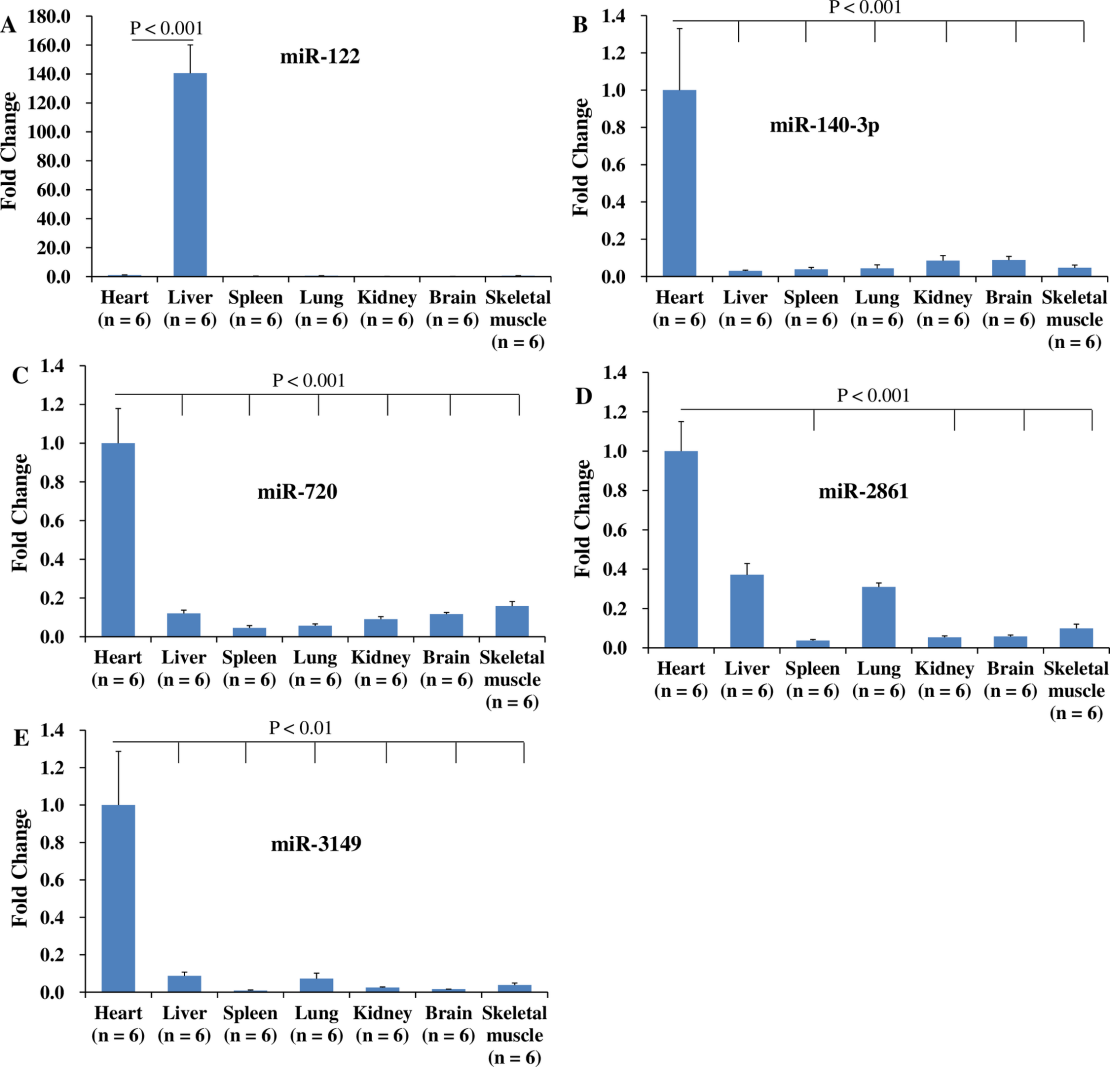
Expression profiles of miR-122, -140-3p, -720, -2861, and -3149 in liver, spleen, lung, kidney, brain, skeletal muscles, and heart of the sham pigs. The expression levels of each miRNA was normalized to its expression levels in heart. MiR-122 was specifically expressed in liver (A), and miR-140-3p, -720, -2861, and -3149 were highly expressed in heart (B to E).

To determine the tissue specificity of miR-122, -140-3p, -720, -2861, and -3149, their expression levels were detected in liver, spleen, lung, kidney, brain, skeletal muscles, and heart of the sham pigs. The results showed that miR-122 was specifically expressed in liver, and miR-140-3p, -720, -2861, and -3149 were highly expressed in heart (Fig 1).

### MiRNAs expression levels in the non-ischemic, border, and ischemic myocardium of the AMI pigs

**Fig 2:**
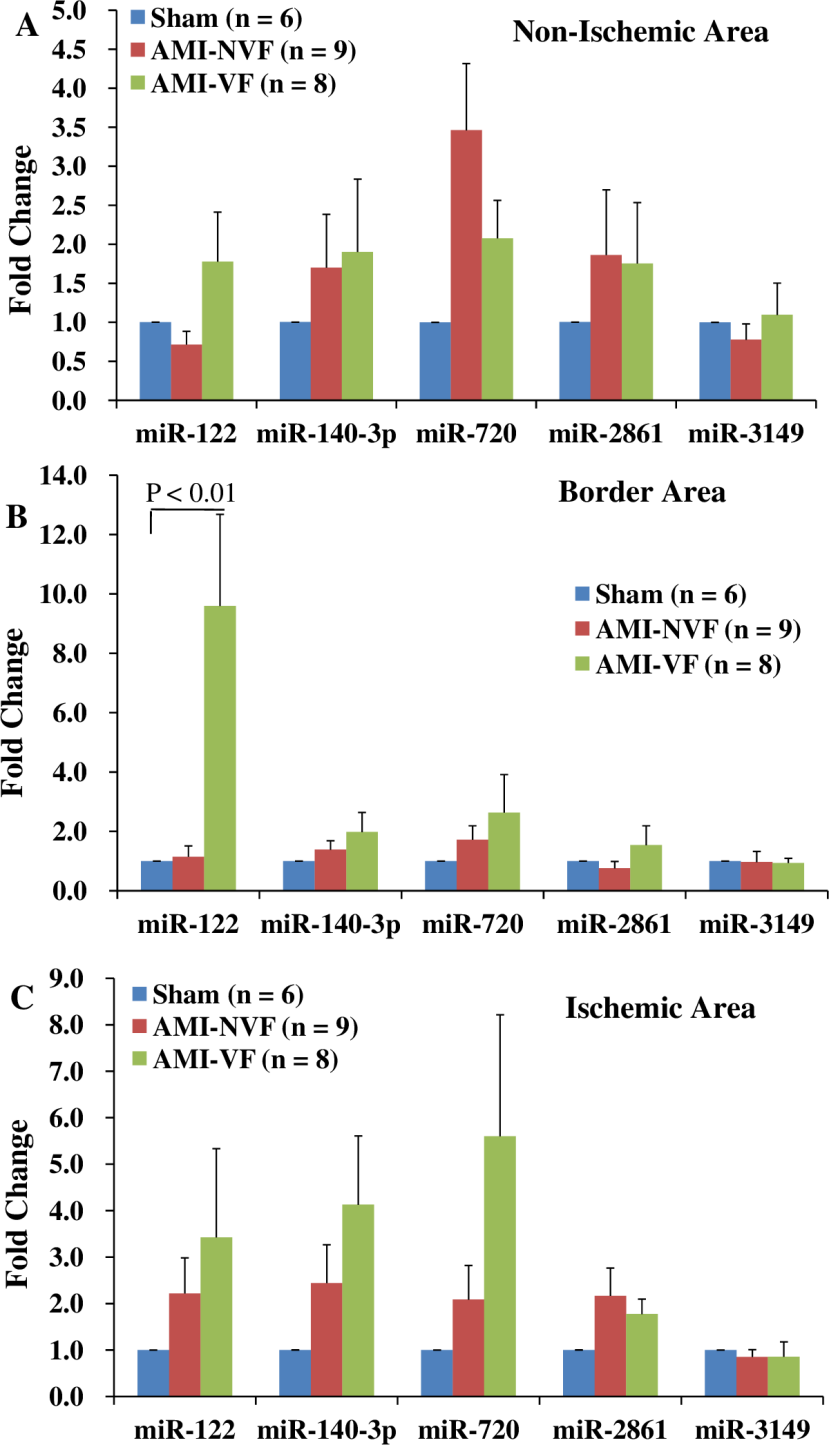
Expression profiles of miR-122, -140-3p, -720, -2861, and -3149 in the non-ischemic, border, and ischemic myocardium of the AMI pigs. The expression levels of each miRNA was normalized to its expression levels in myocardium of the sham pigs. Compared with the sham pigs, miR-122 was increased in the border myocardium of the AMI-VF pigs (B). Abbreviations: AMI = acute myocardial infarction, AMI-VF = acute myocardial infarction with ventricular fibrillation.

To determine whether the elevated plasma miRNAs were originated from the ischemic myocardial cells, these miRNAs were detected in the non-ischemic, border, and ischemic myocardium of the AMI pigs. The results showed that miR-122 was highly expressed in the border myocardium of the AMI-VF pigs (Ct = 30.4±0.9) compared with the sham pigs (Ct = 35.4±0.7) (P < 0.01) (Fig 2B), but no statistical differences of other miRNAs expression levels were detected in the non-ischemic, border, and ischemic areas between the sham and AMI pigs (all P > 0.05) (Fig 2A–2C).

### MiRNAs expression levels in platelets and PBMCs of the AMI pigs and ACS patients

**Fig 3:**
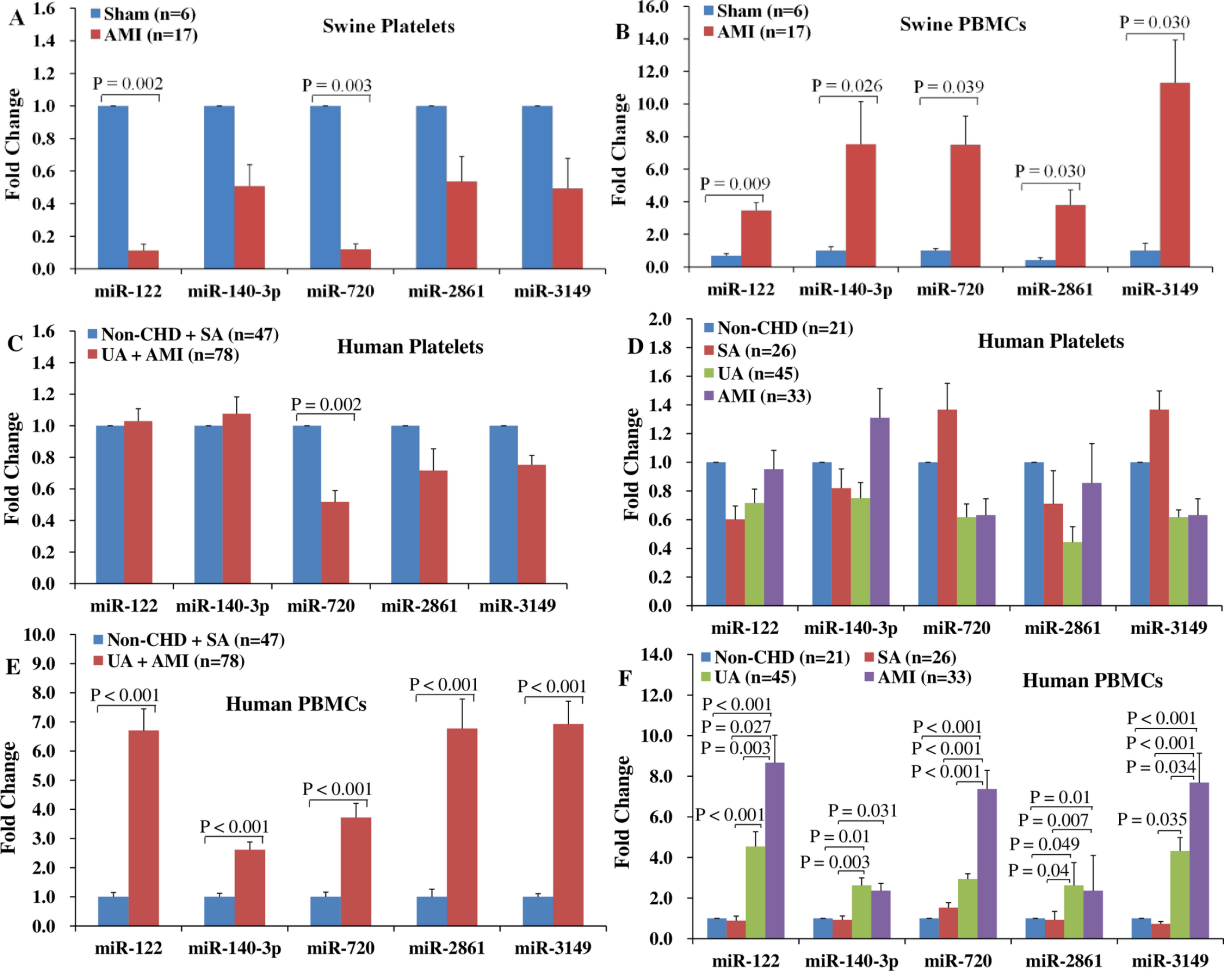
Expression profiles of miR-122, -140-3p, -720, -2861, and -3149 in platelets and PBMCs of the AMI pigs and ACS patients. The expression levels of each miRNA was normalized to its expression levels in the sham pigs or non-CHD patients. Compared with the sham pigs, platelets miR-122 and -720 were decreased (A), and PBMCs miR-122, -140-3p, -720, -2861, and -3149 were increased in the AMI pigs (B). Compared with the non-ACS patients, platelets miR-720 was downregulated (C), and PBMCs miR-122, -140-3p, -720, -2861, and -3149 were upregulated in the ACS patients (E). Furthermore, the expression levels of PBMCs miR-122, -720, and -3149 were higher in the AMI patients than that in the UA patients (F). Abbreviations: PBMCs = peripheral blood mononuclear cells, non-CHD = patients without coronary heart disease, SA = stable angina, UA = unstable angina, AMI = acute myocardial infarction, ACS = acute coronary syndrome, non-ACS = patients without acute coronary syndrome.

Compared with the sham pigs, the expression levels of miR-122 (Ct_sham_ = 34.4±0.9, Ct_AMI_ = 34.8±1.9) and -720 (Ct_sham_ = 19.6±0.8, Ct_AMI_ = 21.6±2.4) in platelets were decreased in the AMI pigs, and the expression levels of miR-122 (Ct_sham_ = 40.3±5.5, Ct_AMI_ = 38.0±2.2), -140-3p (Ct_sham_ = 30.7±0.4, Ct_AMI_ = 30.2±0.3), -720 (Ct_sham_ = 21.9±0.6, Ct_AMI_ = 21.3±0.3), -2861 (Ct_sham_ = 25.7±1.4, Ct_AMI_ = 21.9±0.8), and -3149 (Ct_sham_ = 11.2±0.2, Ct_AMI_ = 10.3±0.3) in PBMCs were increased in the AMI pigs (all P < 0.05) (Fig 3A and 3B).

Compared with the non-ACS patients, platelets miR-720 (Ct_non-ACS_ = 22.2±1.1, Ct_ACS_ = 22.7±1.2) was decreased and PBMCs miR-122 (Ct_non-ACS_ = 34.8±1.2, Ct_ACS_ = 32.2±1.4), -140-3p (Ct_non-ACS_ = 24.6±1.3, Ct_ACS_ = 23.3±1.0), -720 (Ct_non-ACS_ = 20.6±1.3, Ct_ACS_ = 18.6±1.0), -2861 (Ct_non-ACS_ = 21.4±2.1, Ct_ACS_ = 18.3±2.1), and -3149 (Ct_non-ACS_ = 16.4±0.7, Ct_ACS_ = 14.0±1.3) were increased in the ACS patients (all P < 0.01) (Fig 3C and 3E). Furthermore, PBMCs miR-122 (Ct_UA_ = 32.5±1.4, Ct_AMI_ = 31.8±1.4), -720 (Ct_UA_ = 19.1±1.0, Ct_AMI_ = 18.0±0.8), and -3149 (Ct_UA_ = 14.3±1.1, Ct_AMI_ = 13.7±1.3) were increased in the AMI patients compared with the UA patients (all P < 0.05) (Fig 3F).

### MiRNAs expression levels in CECs, monocytes, and lymphocytes of the ACS patients

**Fig 4:**
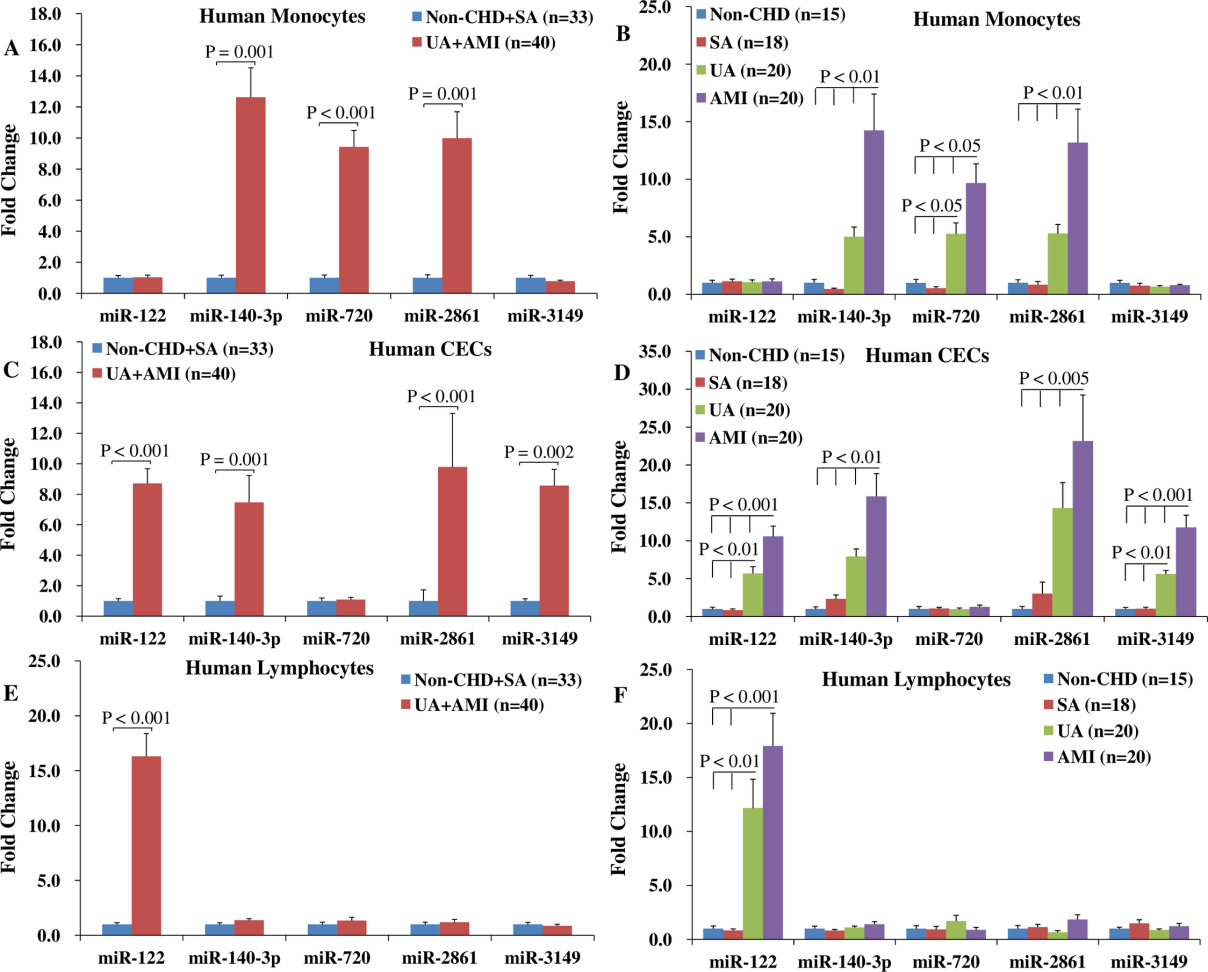
Expression profiles of miR-122, -140-3p, -720, -2861, and -3149 in monocytes, CECs, and lymphocytes. The expression levels of each miRNA was normalized to its expression levels in the non-CHD patients. The expression levels of miR-140-3p, -720, and -2861 in monocytes (A), miR-122, -140-3p, -2861, and -3149 in CECs (C), and miR-122 in lymphocytes (E) were dramatically increased in the ACS patients in contrast to the non-ACS patients. In addition, the expression levels of miR-140-3p, -720, and -2861 in monocytes (B), and miR-122, -140-3p, -2861, and -3149 in CECs (D) were higer in the AMI patients than that in the UA patients. Abbreviations: CECs = circulating endothelial cells, other abbreviations as in Fig 3.

To further identify the origin of the elevated miRNAs in PBMCs, these miRNAs’ expression profiles were detected in monocytes, CECs, and lymphocytes isolated from human PBMCs. The results demonstrated that, compared with the non-ACS patients, miR-140-3p (Ct_non-ACS_ = 26.7±1.01, Ct_ACS_ = 22.5±0.8), -720 (Ct_non-ACS_ = 26.6±0.9, Ct_ACS_ = 22.4±0.6), and -2861 (Ct_non-ACS_ = 26.7±1.3, Ct_ACS_ = 22.7±1.0) in monocytes (Fig 4A), miR-122 (Ct_non-ACS_ = 37.6±1.0, Ct_ACS_ = 34.8±0.7), -140-3p (Ct_non-ACS_ = 34.4±1.3, Ct_ACS_ = 31.6±0.8), -2861 (Ct_non-ACS_ = 26.9±1.8, Ct_ACS_ = 23.4±1.2), and -3149 (Ct_non-ACS_ = 14.5±0.8, Ct_ACS_ = 11.6±0.5) in CECs (Fig 4C), and miR-122 (Ct_non-ACS_ = 38.6±1.0, Ct_ACS_ = 34.6±0.6) in lymphocytes (Fig 4E) were greatly up-regulated in the ACS patients (all P < 0.05).

Furthermore, compared with the UA patients, the expression levels of miR-140-3p (Ct_UA_ = 23.0±0.6, Ct_AMI_ = 22.0±0.6), -720 (Ct_UA_ = 22.6±0.5, Ct_AMI_ = 22.2±0.5), and -2861 (Ct_UA_ = 23.0±0.9, Ct_AMI_ = 22.3±1.1) in monocytes (Fig 4B), and the expression levels of miR-122 (Ct_UA_ = 35.1±0.7, Ct_AMI_ = 34.4±0.6), -140-3p (Ct_UA_ = 31.9±0.6, Ct_AMI_ = 31.2±0.8), -2861 (Ct_UA_ = 23.5±1.3, Ct_AMI_ = 23.1±1.1), and -3149 (Ct_UA_ = 12.0±0.3, Ct_AMI_ = 11.2±0.4) in CECs (Fig 4D) were significantly increased in the AMI patients (all P < 0.05).

## Discussion

Studies have found that elevated miRNAs during ACS come from several origins: skeletal muscle miRNAs (miR-1 [8], -133a [9], and -133b), cardiac-specific miRNAs (miR-208a [9], -208b, -499, and -499-5p [8]), pancreatic miRNAs (miR-375), liver-specific miRNAs (miR-122), brain-enriched miRNAs (miR-34a, -124, -134, and -134-5p [10]) [8–10], and endothelium-enriched miRNAs (miR-221-3p [11], -126, and -423 [12]). However, most of their diagnostic values are controversial in different studies [13]. On the contrary, some of these miRNAs, that come from PBMCs, were more specific for organ ischemia and perhaps related to the underlying mechanisms of ischemia, like endothelial dysfunction, inflammation, or platelets activation.

Endothelial cells is the first barrier to atherosclerosis, while injury and dysfunction of endothelial cells is the first step during the development of atherosclerosis to ACS. CECs, a subpopulation of endothelial cells, are thought to originate from blood vessel walls and are released into the circulation in response to endothelial damage. Variation of CECs count number has been reported to be correlated with the expression levels of endothelin-1 (ET-1), von Willebrand factor (vWF), and tissue factor that reflect the damage of endothelial cells, and has been acknowledged as the only biomarker that can directly assessing endothelial damage in living body, and increased numbers of CECs has been considered to be a prognostic indicator of ACS [14].

MiRNAs have been found to play important roles in senescence [15], inflammatory response, injury and transmigration of endothelial cells [16], atherosclerosis development, plaque formation and rupture [17], and angiogenesis after myocardial infarction [18]. MiR-126, specifically expressed in endothelial cells, was found to reduce leukocyte adherence to endothelial cells, regulate angiogenic signaling and vascular integrity in response to vascular endothelial growth factor (VEGF) and fibroblast growth factor (FGF), and was significantly increased in plasma of AMI patients [12]. MiR-34a, -134 and -134-5p, enriched in endothelial cells, were revealed to be highly expressed in PBMCs of the AMI patients than that in the stable angina patients [10, 19]. MiR-221-3p, a member of antiangiogenic gene-regulating miRNAs family and enriched in the intima layer of endothelial cells of human atherosclerotic vessels, can suppress tube formation, endothelial cell proliferation, migration and angiogenesis by inhibiting endothelial nitric oxide synthase, and facilitate the main pathophysiologic mechanisms of AMI, such as serious endothelial dysfunction, development of coronary atherosclerosis and vulnerable plaques [11, 20]. Recently, Coskunpinar E et al [11] found that miR-221-3p was a promising biomarker for early prediction of AMI and was significantly related to both presence and severity of AMI indicated by Synthax score.

The above study results suggest that endothelial miRNAs are involved in the stability of vascular function and atherosclerotic plaque and ACS development, and miRNAs released by injured endothelial cells might be potential biomarkers for ACS. Our study further found that the expression levels of miR-122, -140-3p, -2861, and -3149 in CECs of ACS patients were significantly higher than that in the non-ACS patients. Recently, upregulated circulating miR-2861 was identified to be significantly correlated with coronary artery calcification in symptomatic patients of obstructive coronary disease [21]. Therefore, our study results suggest that these miRNAs, released by injured vascular endothelial cells, have the potential to be biomarkers for ACS diagnosis and deserve further study.

Monocytes are activated during atherosclerosis development in response to chemoattractants and specific adhesion molecules presented by endothelial cells, and critically drive atherosclerotic lesion formation. Circulating monocytes are divided into classical CD14+ monocytes, comprising 85–95% of circulating monocytes, and ‘tissue-resident’ CD14+CD16+ monocytes, which can be subdivided into intermediate CD14++CD16+ monocytes and nonclassical CD14+CD16++ monocytes [22]. CD14+CD16+ monocytes are elevated in the patients with unstable angina and plaque rupture compared with the stable angina patients, and elevation of CD14++CD16+ monocytes is an independent predictor of cardiovascular events in subjects with high risk of coronary disease [23].

Studies have found that miRNAs in monocytes play important roles in the development of atherosclerosis, inflammatory response, plaque rupture, and ACS. MiR-155, enriched in CD14^+^ monocytes, was closely related with inflammation and could pleiotropically regulate atherosclerosis [24]. MiR-155 was reported to be upregulated in human carotid plaque, and could prompt the inflammatory response during atherosclerosis by repressing B-cell leukemia/lymphoma 6 (Bcl6) in macrophages [25]. However, another study revealed that miR-155 was significantly down-regulated in circulation of the CHD patients [26], and acted as an anti-inflammatory and atheroprotective miRNA, by inhibiting atherosclerotic plaque development, maintaining plaque stability, and decreasing monocyte subset differentiation [27]. Recently, miR-155-5p was reported to be upregulated after plaque rupture for 0.5 h and 1 h [25], and miR-124, highly expressed in monocyte/macrophage cells, was as well a promising biomarker for AMI diagnosis [10, 19], suggesting that monocytes-released miRNAs could be useful for AMI early diagnosis.

Thus, these studies demonstrated that miRNAs affect mononuclear macrophages-mediated pathological process, such as lipid uptake, inflammatory factors and inflammation [28], atherosclerotic plaque formation and rupture [17], and angiogenesis after myocardial infarction [18]. In addition, the anti-atherosclerosis effects of statins and pioglitazone relate to the regulation of monocytes miRNAs [29, 30], suggesting that monocytes-released miRNAs during ACS might be important indicators of ACS. Our study demonstrated that the expression levels of miR-140-3p, -720, and -2861 in monocytes of the ACS patients were significantly higher than that in the non-ACS patients, suggesting that these miRNAs were originated from the activated monocytes during ACS, and these miRNAs have the potential to be biomarkers for ACS diagnosis and deserve further study.

Our study found that miR-122 and -3149 were mainly released by endothelial cells, miR-720 was mainly come from monocytes, and miR-140-3p and -2861 were originated from both endothelial cells and monocytes. MiR-122, initially discovered as a liver specific miRNA, played crucial roles in lipid metabolism [31], and was associated with the presence and severity of CHD in hyperlipidemia patients [32]. In the healthy heart, miR-122 was expressed at very low levels or undetectable [8], but increased in the border and ischemic areas of the AMI mice [8], in plasma of the patients with acute heart failure [33], and in plasma of the porcine cardiogenic shock model [34]. Recent evidences showed that miR-122-5p played important roles in breast cancer cell progression, osteoporotic fracture, and liver injury [35]. miR-3149, located at Chromosome 8—NC_000008.11, was down-regulated in gastric cancer [36]. As cancers, bone and bone marrow, and liver tissues are all with high amount of micro-vessels, this can explain why miR-122 and -3149 were mainly come from endothelial cells.

MiR-720 is an important regulator of T cell proliferation, regulating the cell cycle at both early and later stages during T cell activation [37]. MiR-720 was upregulated in a variety of tumors including colorectal and bladder cancers, malignant melanoma, renal cell carcinoma and multiple myeloma, and played an important role in tumor formation, invasion, and migration [38]. Besides, upregulation of miR-720 in HBV-specific T lymphocytes played a critical role in host immunity during chronic HBV infection [37]. Recently, miR-720 was found to be down-regulated in tumour associated macrophages and M2-polarized macrophages, and played a role in tumour immune microenvironment [39]. This may explain why miR-720 was mainly released by monocytes and macrophages in our present study.

MiR-140 is expressed in numerous tissues and cell types including brain, breast, lung, colon, ovary and testis, and acts as a potential tumor suppressor in cutaneous squamous cell carcinoma, basal cell carcinoma, osteosarcoma, ovarian cancer, colon cancer, lung squamous cell carcinoma, and breast cancer [40]. MiR-140-3p has been reported to be highly expressed in blood of the CHD patients [41] and in PBMCs of the diabetes mellitus patients [42]. MiR-2861 was up-regulated in basal cells [43] and papillary thyroid carcinoma [44], and played a positive role in osteoblast differentiation and chordoma development [45]. Whereas, miR-2861 was greatly decreased in basal cell carcinoma and cervical cancer, and negatively correlated with advanced tumor stage and lymph node metastasis [46]. Overexpression of miR-2861 suppressed cervical cancer cell proliferation and invasion, and enhanced apoptosis [46]. As tumor, full of micro-vessels, is also considered to be a kind of immune disease and monocytes/macrophages plays an immportant role in tumor immune microenvironment, and CHD and diabetes mellitus are characterized with injury of endothelial cells. Therefore, these studies suggest that miR-140-3p and -2861 are highly expressed in both endothelial cells and monocytes/macrophages.

In conclusion, the elevated plasma miR-122, -140-3p, -720, -2861, and -3149 during early ACS, were mainly originated from CECs and monocytes.
